# Supplementary material for: Sporadic low-velocity volumes spatially correlate with shallow very low frequency earthquake clusters
Source: Nat Commun. 2017 Dec 11;8:2048. doi: 10.1038/s41467-017-02276-8 (PMC5725571; doi:10.1038/s41467-017-02276-8)
Supplement: Supplementary file 1 — Supplementary Information [file 41467_2017_2276_MOESM1_ESM.pdf]

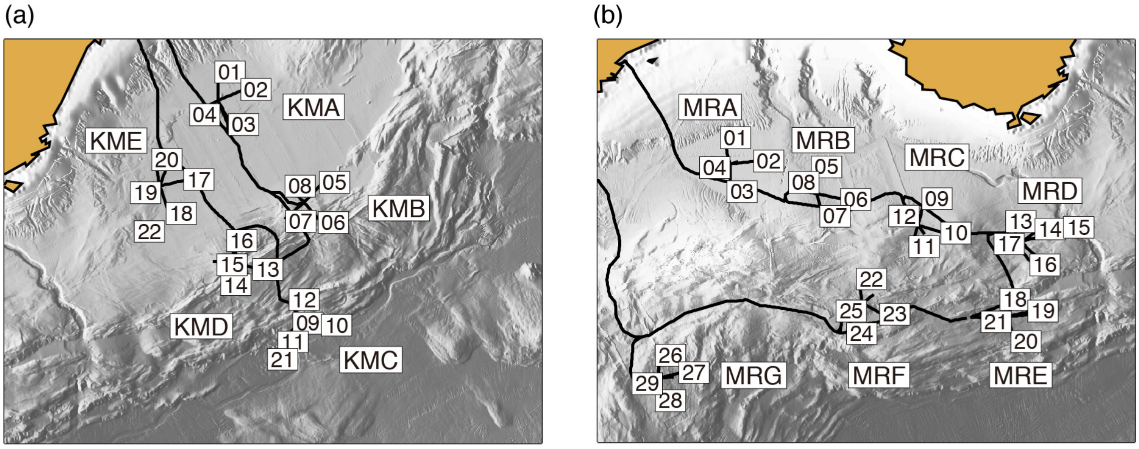

Supplementary Figure 1 | Names of nodes and stations. Squares indicate station locations for (a) DONET1 and (b) DONET2. Black lines represent cables.

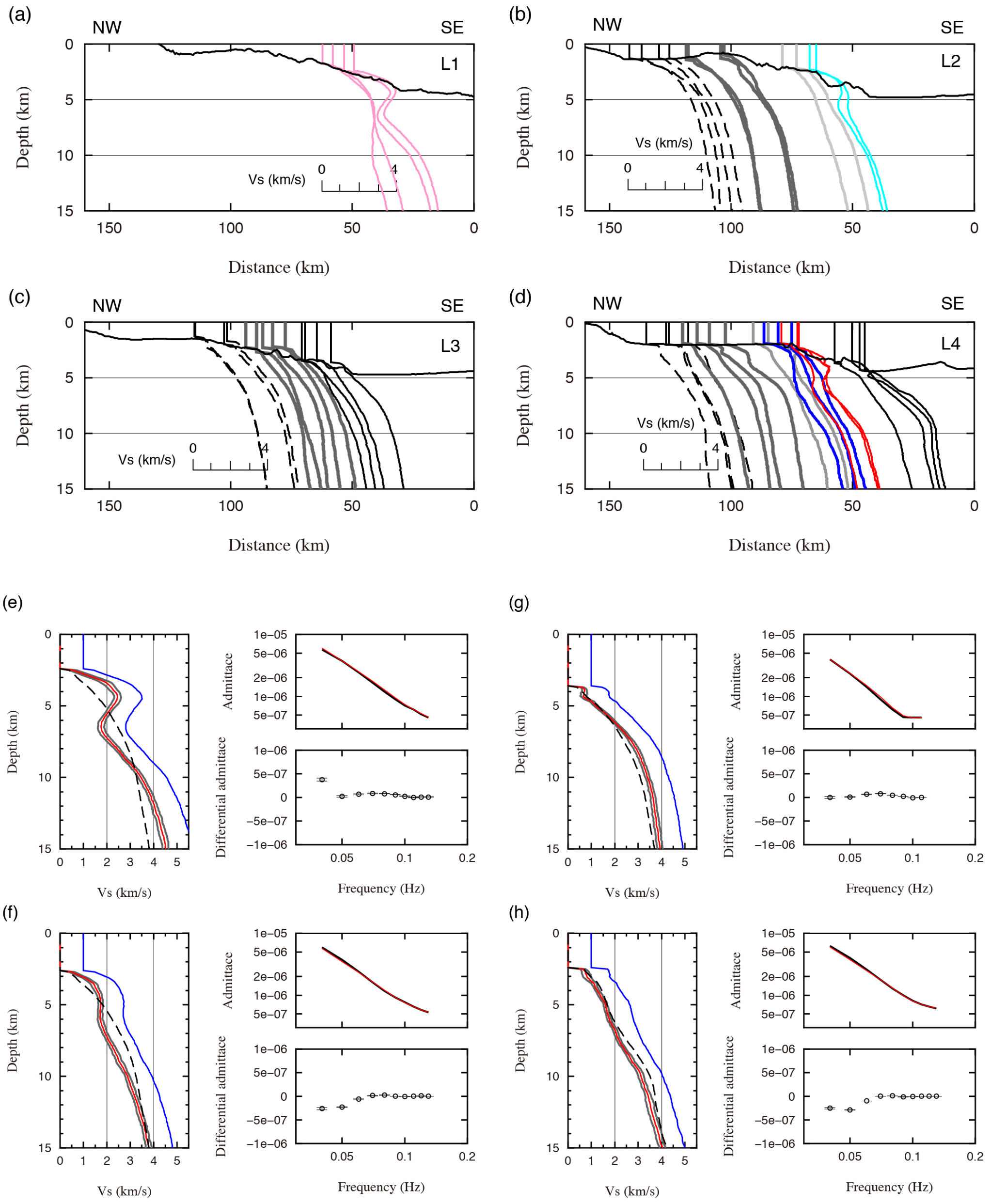

Supplementary Figure 2 | *Vs* profiles at each station. (a) Same as Fig. 2, but for e-RAs. (b)–(d) Same as panel a, but for L2–L4. (e) (Left) Obtained *Vs* profile at MRG27 in L1 (red) and its standard deviation (grey) estimated using 50 en-RAs. Dashed line indicates initial velocity model. Blue line represents *Vs* profile from e-RAs, in which a shift of +1 km s^{-1} is added to the original one for comparison with red line. (Right upper panel) Black and red lines are observed and inverted RAs, respectively, averaged over 50 RAs in inversion. Dashed line corresponds to RA from the unperturbed original velocity model. (Right lower panel) Circles and error bars represent differences between observed and inverted RAs. The uncertainty is the standard error multiplied by 2. (f)–(h) Same as panel e, but for MRF23 in L2, MRE20 in L3, and KMB06 in L4

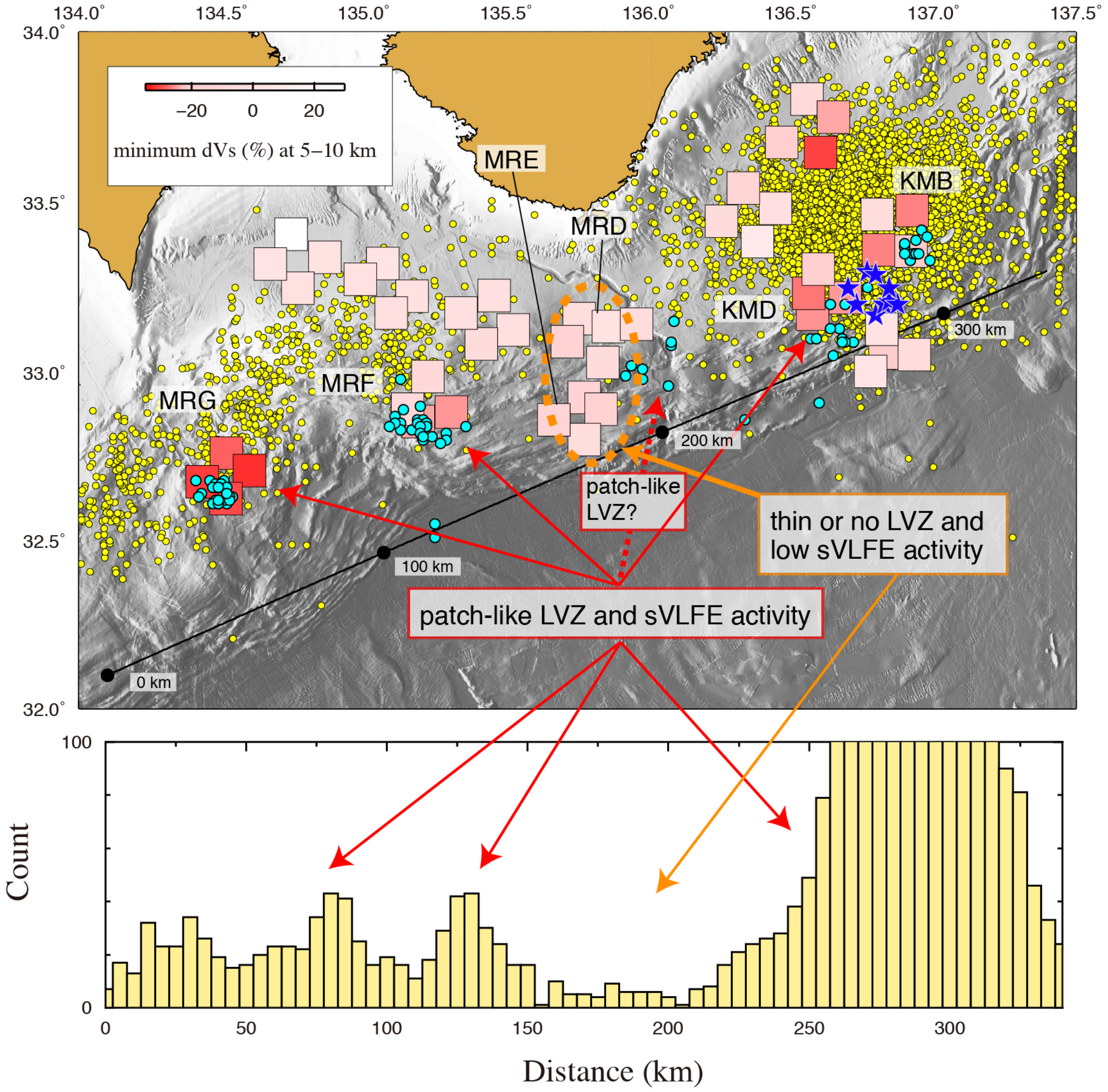

Supplementary Figure 3 | Spatial relationship between LVZ and sVLFE determined by land and seafloor records. (top) The intensity of red in the squares indicates the minimum *dVs* in the depth range of 5–10 kmbsl. Locations of squares correspond to station locations. Yellow[27] and pale blue circles[29] and blue stars[23] show epicentres of sVLFEs from the three catalogues. (bottom) An along-strike histogram for the number of VLFEs with a distance increment of 5 km along a solid line in the top panel, in which the epicentres are projected perpendicularly to the direction of the line, and the number of sVLFEs at each 5 km bin is counted.

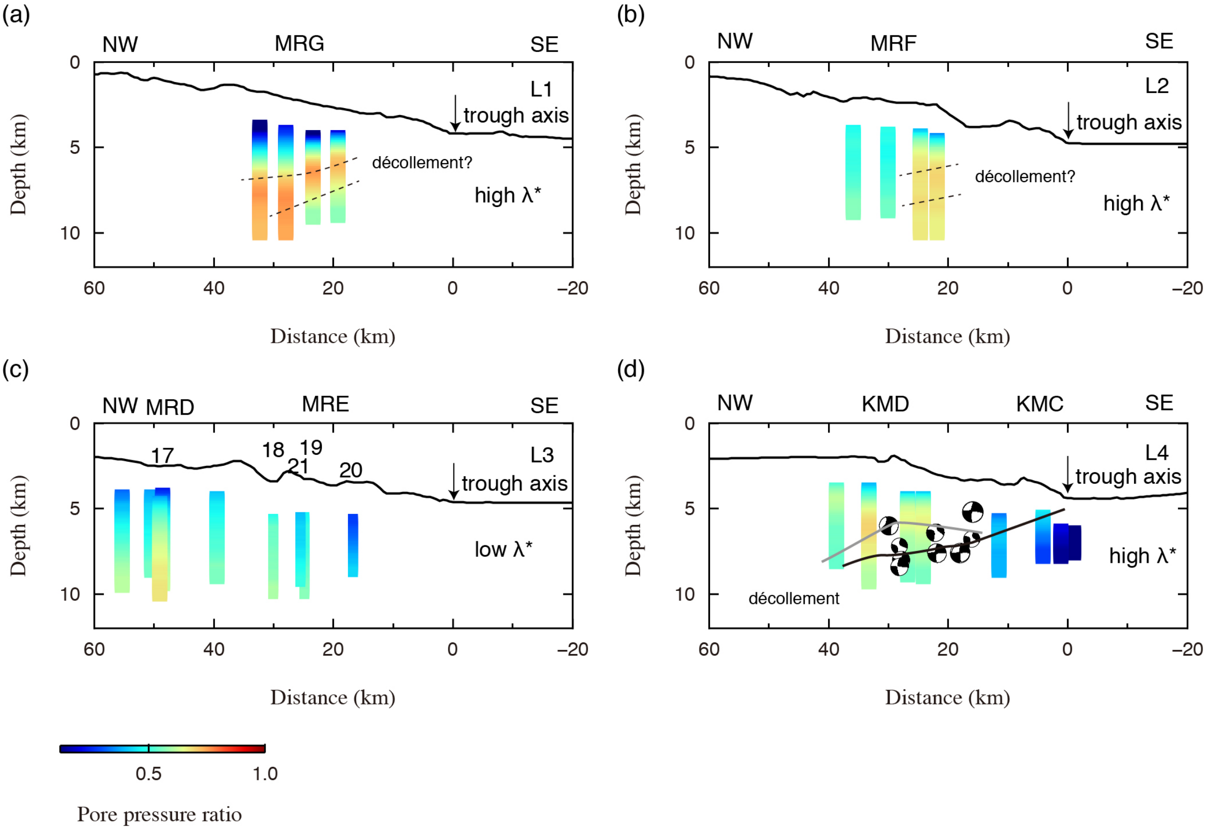

Supplementary Figure 4 | Pore pressure ratio profiles for each line. Pore pressure ratio profiles for stations at (a) node MRG in L1, (b) node MRF in L2, (c) nodes MRF and MRE in L3, and (d) nodes of KMC and KMD in L4. Numbers in panel c are MRE station codes for each *Vs* profile. Black[17] and gray[18] lines in panel d represent the décollement according to previous studies, and dashed line in panels a–b indicates possible location of décollement.

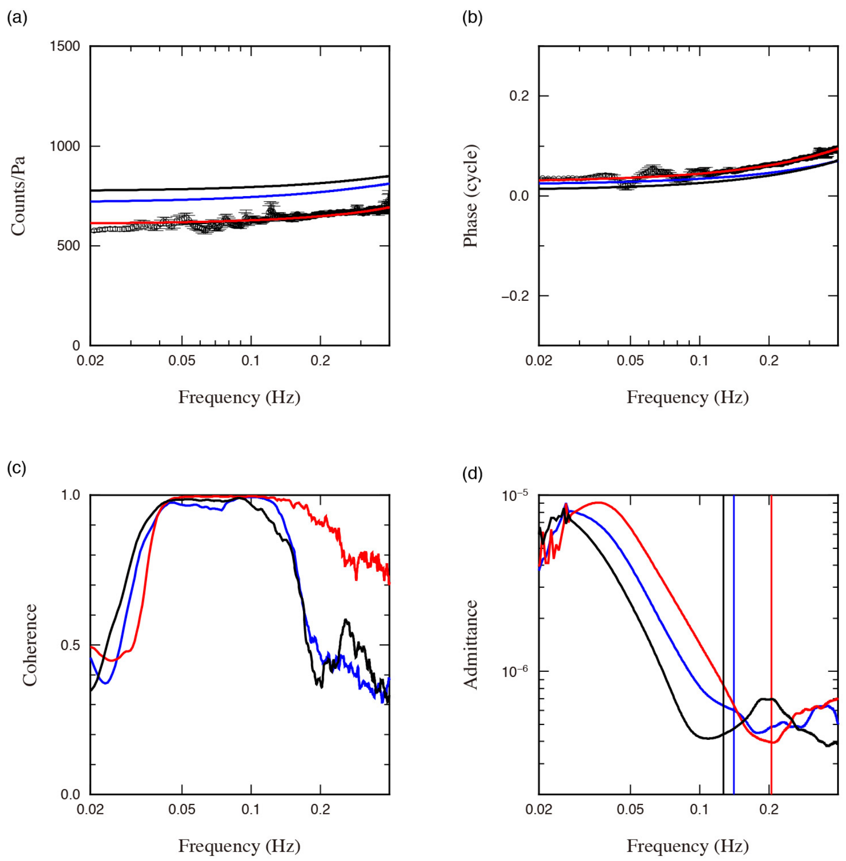

Supplementary Figure 5 | Examples of transfer functions at three stations. (a) Amplitude transfer function between DPG and APG for stations KMC12 (black), MRA01 (red), and KMB06 (blue). Circles with error bars for MRA01 indicate original data used for fitting quadratic function. (b) Phase between DPG and APG. Circles with error bars for MRA01 indicate original data used for fitting the quadratic function. (c) Coherence between displacement and the corrected pressure observed by DPG. (d) Values of e-RAs calculated from displacements and corrected pressure fluctuations. Vertical lines represent fr_2 for each RA.

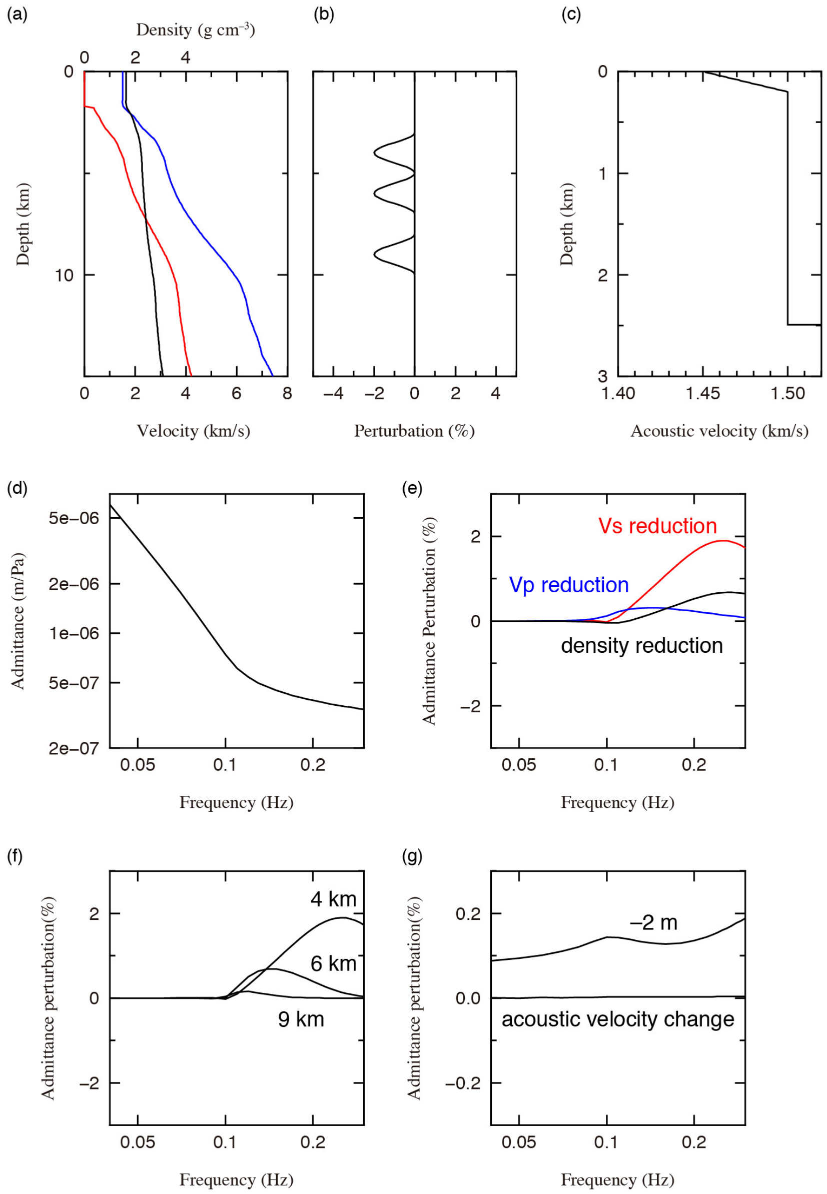

Supplementary Figure 6 | Synthetic test of RA sensitivity for physical parameters. (a) Blue, red, and black lines show original *Vp*, *Vs*, and density profiles, respectively. (b) Perturbations as a function of depth (panel f; model D in Supplementary Table 2). (c) Acoustic velocity profile with a reduction at upper 0.2 km depth (model F in Supplementary Table 2). (d) RA for original velocity and density profiles. (e) RA perturbations from RA in panel d for *Vp* (blue), *Vs* (red), and density (black) reductions (models A–C in Supplementary Table 2). (f) Same as panel e, but for depth-dependent *Vs* changes (panel b; model D in Supplementary Table 2). (g) Same as panel e, but for a -2 m change in water depth (model E in Supplementary Table 2) and an acoustic velocity change (panel c; model F in Table 2).

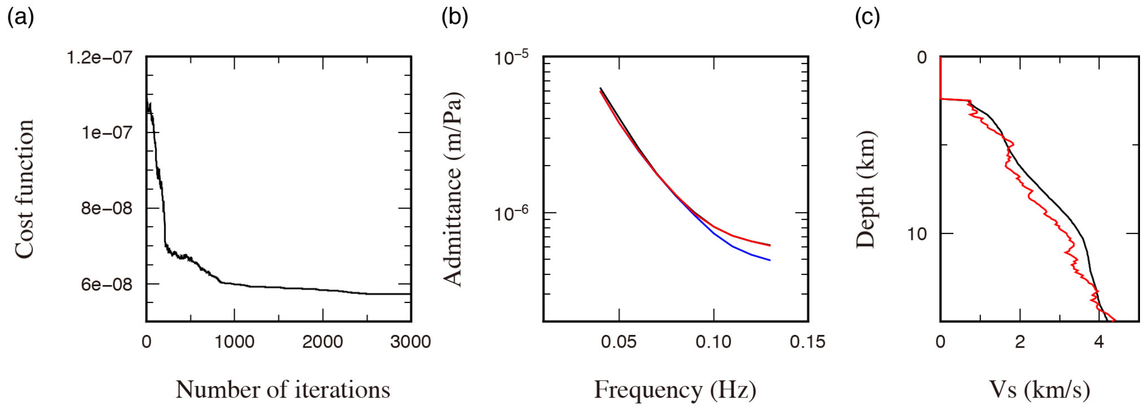

Supplementary Figure 7 | Examples from one iteration sequence for KMB06. (a) Cost function sequence as a function of iteration steps. (b) Observed (black) and inverted (red) RAs, and an RA calculated from the initial velocity model (blue). (c) Obtained (red) and initial (black) *Vs* profiles from one iteration sequence.

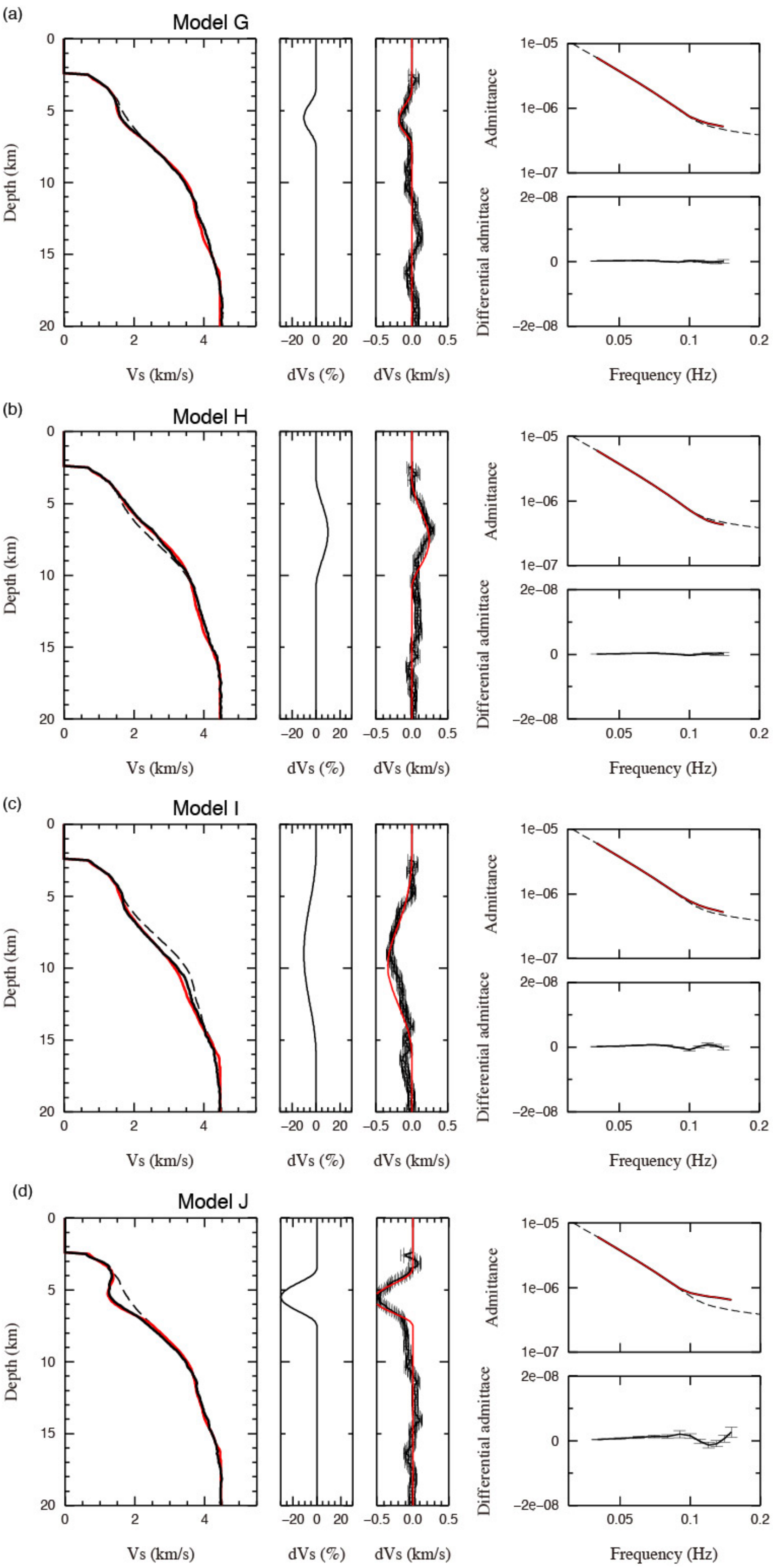
(a)
Model G
(b)
Model H
(c)
Model I
(d)
Model J
Depth (km)
Vs (km/s)
dVs (%)
dVs (km/s)
Admittance
Differential admittace
Frequency (Hz)
1e−05
1e−06
1e−07
2e−08
0
−2e−08
0.05
0.1
0.2
−20
20
−0.5
0.0
0.5

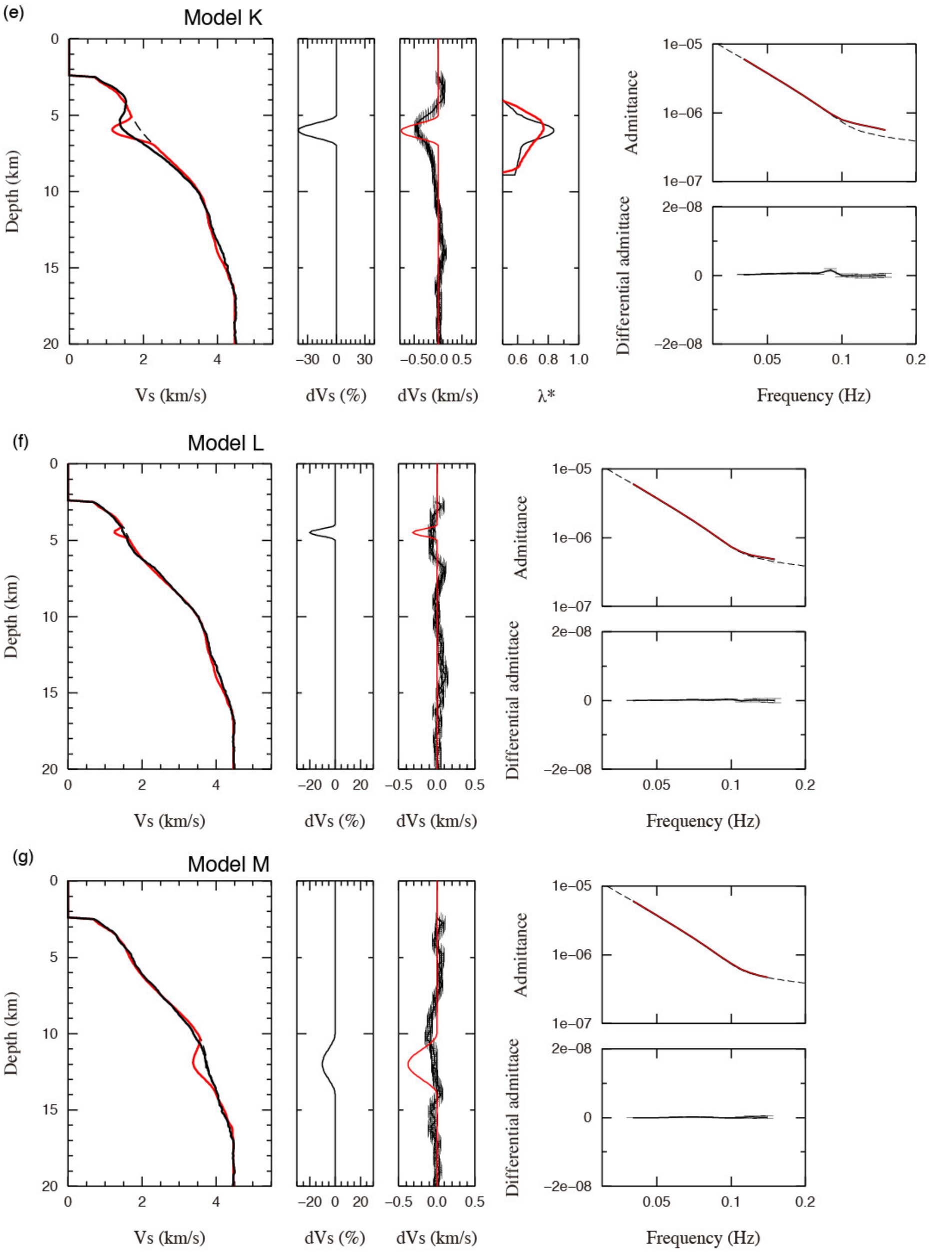

Supplementary Figure 8 | Synthetic tests for employed inversion technique. (a) (Left) Black, red and dashed lines indicate obtained result from inversion, perturbed (correct answer), and initial velocity models for model G (Supplementary Table 3). Perturbations in percent and km s^{-1} are displayed in second and third columns. Obtained results and errors are also displayed in right column. (Right) Black and red lines indicate RAs for obtained and perturbed (correct answer) velocity models (top) and their difference with error bars (bottom). (b–f) Same as (a), but for models H–M (Supplementary Table 3). The panel (e) contains the pore pressure ratio profile (black: correct answer, red: obtained result).

Supplementary Table 1. Number of events and fr_1, fr_2 at each station

DONET1 station	N_1† (event)	N_2† (event)	fr_1 (Hz)	fr_2 (Hz)	DONET2 station	N_1† (event)	N_2† (event)	fr_1 (Hz)	fr_2 (Hz)
KMA01	44	597	0.09	0.15	MRA01	56	89	0.14	0.21
KMA02	44	688	0.1	0.15	MRA02	47	85	0.12	0.2
KMA03	43	604	0.09	0.14	MRA03	64	103	0.12	0.24
KMA04	43	650	0.1	0.14	MRA04	56	88	0.14	0.2
KMB05	43	576	0.08	0.15	MRB05	53	104	0.14	0.2
KMB06	43	703	0.09	0.14	MRB06	48	86	0.1	0.2
KMB07	43	285	0.09	0.14	MRB07	36	33	0.12	0.23
KMB08	43	472	0.09	0.16	MRB08	56	67	0.13	0.24
KMC09	43	936	0.07	0.12	MRC09	36	106	0.09	0.17
KMC10	43	496	0.09	0.12	MRC10	36	101	0.1	0.16
KMC11	43	638	0.07	0.13	MRC11	56	173	0.09	0.15
KMC12	43	808	0.08	0.12	MRC12	36	70	0.11	0.19
KMD13	43	398	0.08	0.12	MRD13	24	137	0.08	0.14
KMD14	43	512	0.09	0.13	MRD14	28	138	0.08	0.13
KMD15	43	490	0.08	0.16	MRD15	24	115	0.08	0.14
KMD16	43	609	0.08	0.16	MRD16	20	113	0.08	0.11
KME17	39	484	0.08	0.16	MRD17	20	136	0.07	0.12
KME18	39	471	0.08	0.16	MRE18	49	287	0.07	0.11
KME19	39	415	0.08	0.17	MRE19	36	243	0.06	0.11
KME20	39	426	0.08	0.16	MRE20	24	157	0.07	0.11
					MRE21	36	223	0.09	0.11
					MRF22	35	106	0.11	0.14
					MRF23	35	202	0.07	0.13
					MRF24	24	134	0.08	0.13
					MRF25	48	187	0.08	0.13
					MRG26	36	97	0.1	0.16
					MRG27	28	131	0.08	0.13
					MRG28	24	122	0.08	0.13
					MRG29	36	150	0.08	0.16

†N_1 and N_2 are the number of events for estimating DPG/APG and e-RA, respectively.

Supplementary Table 2. Physical parameter perturbations for calculations of RA sensitivity

	Vp (%)	*Vs* (%)	Density (%)	Depth (km)	Water depth change (m)
Model A	–2	0	0	4±1	0
Model B	0	–2	0	4±1	0
Model C	0	0	–2	4±1	0
Model D	0	–2	0	4±1, 6±1, 9±1	0
Model E	0	0	0	–	–2
Model F	–3.3	0	0	0–0.2	0

Supplementary Table 3. *Vs* perturbations for inversion tests

	Max. perturbation in *Vs* (%)	Center depth (km)	Half thickness from the center depth (km)
Model G	–10	5.5	2.0
Model H	+10	7.0	2.5
Model I	–10	9.0	7.0
Model J	–30	5.5	2.0
Model K	–40	6.0	1.0
Model L	–20	4.5	0.5
Model M	–10	12.0	2.0
